# Associations of Pre-Pregnancy BMI, Gestational Weight Gain and Maternal Parity with the Trajectory of Weight in Early Childhood: A Prospective Cohort Study

**DOI:** 10.3390/ijerph16071110

**Published:** 2019-03-28

**Authors:** Tingting Sha, Xiao Gao, Cheng Chen, Ling Li, Qiong He, Xialing Wu, Gang Cheng, Qianling Tian, Fan Yang, Yan Yan

**Affiliations:** Department of Epidemiology and Medical Statistics, Xiangya School of Public Health, Central South University, Xiangya Road 110, Changsha 410078, China; tingtingsha@csu.edu.cn (T.S.); 18670321975@163.com (X.G.); chengchen_8493@126.com (C.C.); 18674824070@163.com (L.L.); qqniuniu0525@163.com (Q.H.); wuxl9404@163.com (X.W.); gangcheng@csu.edu.cn (G.C.); 15274979382@163.com (Q.T.); yyangff009@163.com (F.Y.)

**Keywords:** child weight, parity, pre-pregnancy body mass index, gestational weight gain, trajectory

## Abstract

Background: The association of maternal parity, pre-pregnancy body mass index (BMI) and gestational weight gain (GWG) with childhood weight status has been well studied; however, little is known about these factors with respect to the rate of weight changes in early childhood. Methods: This study was based on a prospective longitudinal study. The follow-up surveys were conducted at the ages of 1, 3, 6, 8, 12, and 18 months. Child weight was investigated twice at each wave. Data on maternal parity, pre-pregnancy weight and height were collected at baseline. The latent growth curve model was used to examine the effects of interested predictors on the trajectory of weight in early childhood. Results: Finally, 893 eligible mother-child pairs were drawn from the cohort. In adjusted models, multiparas were associated with higher birth weight (β = 0.103) and slower weight change rate of children (β = −0.028). Pre-conception BMI (β = 0.034) and GWG (β = 0.014) played important roles in the initial status of child weight but did not have effects on the rate of weight changes of the child. Conclusions: Multiparous pregnancy is associated with both higher mean birth weight and slower weight-growth velocity in early childhood, while pregravid maternal BMI and GWG are only related to the birth weight.

## 1. Introduction

In China, the prevalence of overweight/obesity in women aged 18–44 years has increased from 19.9% to 33.5% from 1992 to 2010 [1]. Excessive gestational weight gain (GWG), in parallel to the rapid increase of overweight/obesity, has also been a growing global epidemic concern [2]. Data from recent studies reported that nearly one-third of women had excessive GWG in pregnancy [3]. Emerging evidence has suggested that increased levels of body mass index (BMI) at conception and excessive GWG might be associated with the adverse pregnancy outcomes, such as gestational diabetes mellitus, gestational hypertension, and maternal postpartum weight retention [4,5]. In addition, their fetuses are exposed to an unfavorable in utero metabolic environment [6], to increased levels of chronic inflammation, insulin resistance [7], glucose and fatty acids [8,9], resulting in a greater risk of obesity in offspring and poor health outcomes over the short- and long-term [4,10].

Increasing obesity trends that are observed early in fetal growth [10], offspring, even in childhood [11] might contribute to the harmful changes in the environment which children are born and grow up in [12]. This increasing evidence has led to the hypothesis that maternal pre-pregnancy BMI or GWG status is associated with lifelong consequences in offspring, childhood, and, possibly, over successive generations.

Factors related to the children’s weight such as GWG, pre-pregnancy BMI, and parity, have been extensively studied. However, several gaps in the evidence base challenge the existing results. Most of the previous studies have concentrated on effects of pre-pregnancy BMI or GWG on fetal growth, childhood, or adolescence weight/BMI status; however, little is known about the longitudinal associations of pre-pregnancy BMI or GWG with the rate of development of child weight. Moreover, a limitation is that pre-pregnancy weight used in previous studies mostly relied on self-reported weight, resulting in the underestimation of BMI and overestimation of GWG [13]. In addition, the association between maternal parity and children’s weight has yielded conflicting findings in previous studies [14], with several [15,16], but not all [17], suggesting that multiparity might be associated with the rates of accelerated infant growth and the levels of childhood BMI.

Although childhood obesity-prevention efforts have been predominantly school-based, estimates show that 41 million children younger than 5 years of age globally have been already affected by overweight and obesity when they started school [18]. Emerging evidence suggests that the duration of pregnancy and early life, particular in the “first 1000 days” of childhood, are critical periods for the obesity development of children [19,20]. Therefore, it is helpful to discover children who are susceptible to overweight and obesity in early childhood by identifying the potential risk factors for children’s weight, especially understanding the roles of GWG, pre-pregnancy BMI, and maternal parity.

The latent growth curves model (LGCM) is one of the advanced analytical methods which can create random intercepts and slopes to depict the different trajectories over time. LGCM can not only model the intra-individual and inter-individual change but also it permits exploration of the antecedents and consequences of change. Therefore, based on a community-based cohort study, the present study adopted the LGCM to describe the longitudinal developmental trajectories of child weight in first 18 months of life and examined the association of the pre-pregnancy BMI, GWG, and maternal parity with the weight status and the rates of weight changes of children.

## 2. Materials and Methods

### 2.1. Study Population

This study was based on an in-progress community-based cohort study, which was conducted in Changsha, China. The baseline survey was conducted at three Community Health Service Centers (CHSCs) of Kaifu District of Changsha from January 2015 to December 2015. The follow-up surveys were conducted at infants’ regular check-ups at the ages of 1, 3, 6, 8, 12, and 18 months. The sample in our final analysis only included respondents who were permanent residents in Kaifu District, delivered live-born babies during study period, provided complete health care records in the CHSCs, had no history of mental illnesses or brain diseases, completed the follow-up surveys, and agreed to participate and signed the written informed consents, and excluded mothers with multiple births, conceived via assisted reproductive techniques, and without antenatal care information. Detailed eligibility requirements of subjects are available in the previous study [21].

### 2.2. Measurement of Maternal Pre-Pregnancy BMI, GWG, and Parity

Maternal weight and height were measured by trained nurses, using the same standardized techniques (RGZ-120-RT, Wuxi Weighing Apparatus Co., Wuxi, China) with participants wearing light clothing and no shoes. Weight was measured within the first prenatal care visit occurring within 13 weeks of gestation as a proxy for pre-pregnancy weight. According to the Working Group on Obesity in China, pre-pregnancy BMI was calculated as kg/m^2^ and classified into four categories: underweight (BMI < 18.5 kg/m^2^), normal weight (18.5 kg/m^2^ ≤ BMI < 24.0 kg/m^2^), overweight (24.0 kg/m^2^ ≤ BMI < 28.0 kg/m^2^), and obesity (BMI ≥ 28.0 kg/m^2^) [1].

GWG was calculated as the difference between pre-pregnancy weight and maternal delivery weight. Considering the recommendations of the 2009 Institute of Medicine (IOM) [22], GWG was defined as the following three categories: inadequate, adequate, and excessive, on the basis of pre-pregnancy BMI. The IOM-recommended GWG is 12.5–18 kg for underweight, 11.5–16 kg for normal weight, 7–11.5 kg for overweight, and 5–9 kg for obesity.

Data for maternal parity were collected through the child’s maternity card from the community health management information system, and were divided into two categories: primiparous and multiparous.

### 2.3. Measurement of Children’s Weight

At regular check-ups, children’s weight was tested twice on a digital scale with a precision of 0.1 kg by the trained nurses with light clothing, no shoes, and no caps. Child weight was the average of two measurements. Birth weight was extracted from the child’s maternity card.

### 2.4. Measurement of Covariates

Based on the previous literature [23,24], the following potential confounding factors were identified and assessed: (1) socio-demographic factors: maternal age (<25, 25–29, 30–34, or ≥35 years), parental educational attainment (≤junior school, senior school or ≥college) and household income (<2000, 2000–5000, 5001–10,000, or >10,000 yuan); (2) children’s characteristics: gender (male or female) and gestational week of birth (<28, 28–36, 37–42 or >42 weeks), and (3) the children’s feeding practices: provided any breastfeeding or formula for children. Except for feeding practices, other covariates were collected at the baseline survey within 15 days after delivery. Breastfeeding practices were assessed by WHO-recommended definitions.

Current daily infant-feeding practices were collected by asking their mothers at 1 month, 3 months, 6 months, 8 months, 12 months, and 18 months postpartum through face–to-face interviews. Mothers were asked by the following questions to measure the infant-feeding practices: (1) Whether they engaged in any breastfeeding or stopped breastfeeding for infants at a given time point; (2) whether they provided any formula for infants at a given time point.

### 2.5. Data Analysis

The descriptive statistics were conducted using SAS version 9.4 (SAS Institute, Cary, NC, USA). LGCM analyses and multiple imputations were performed using Mplus, version 7.0 (Muthén & Muthén, Los Angeles, CA, USA). All tests were two-tailed and performed at a level of significance of α = 0.05.

#### 2.5.1. Descriptive Statistics

Continuous variables were presented as mean (standard deviation (SD)) if normally distributed, median (interquartile range (IQR)) if not, and categorical variables were presented as numbers (proportions). Chi-square tests and t-tests were used to examine the differences in the missing and non-missing data group. The one-way repeated ANOVA analyses or t-tests were applied to assess the changes in children’s weight by pre-pregnancy BMI, GWG, and maternal parity.

#### 2.5.2. Latent Growth Curve Model

LGCMs were used to investigate the association of trajectories of child weight aged 0–18 months. The trajectories of weight change were modeled with two latent variables: Latent intercept growth factor, representing the initial status of the child weight; and latent slope growth factor, reflecting the rate of the weight change. The loadings from the intercept factor to each of the repeated measures were set to the fixed values of 1.0 and the slope factor loadings allowed flexibility settings. In this study, we modeled the trajectory of children’s weight based on three assumptions to identify the most suitable model. First, for a linear change assumption of child weight, the slope factor loading was set at 1, 2, 3, to 6 for 1-month-old, 3-month-old, 6-month-old, to 18-month-old respectively. Second, with quadratic change assumption, an additional quadratic slope factor with appropriate factor loadings needed to be added to represent the quadratic term. For example, the quadratic slope factor loading was set at 1 for the first follow-up, 4 for the second follow-up, and so on. Thirdly, a more flexible change assumption was modeled by setting some of the factors free for the subsequent follow-up measures.

The following indices were used to assess the goodness of model fit: χ^2^ statistic, Tucker-Lewis index (TLI) ≥ 0.90, comparative fit index (CFI) ≥ 0.90, standardized root mean square residual (SRMR) ≤ 0.50, and root mean square error of approximation (RMSEA) ≤ 0.08 [25]. One hypothesis in this research was that data were missing at random. Statistical analyses for LGCM were conducted with the robust maximum likelihood estimator method which provided the robust estimates in the presence of non-normality and non-independence of observations [26].

Finally, we performed with multiple imputations to evaluate the evidence of bias from the participants with missing data. We generated 50 imputed data sets. LGCM was repeated using each of the augmented data sets, and parameter estimates were averaged across the 50 analyses.

### 2.6. Ethics Approval and Consent to Participate

This study was conducted under the approval of the independent Ethics Committee (EC) of clinical pharmacology institute of Central South University (CTXY-130041-3-2).

## 3. Results

### 3.1. Descriptive Analysis

A total of 1286 infants were born during the baseline survey. Two-hundred and sixty-five mother-child pairs were not permanent residents, and 45 refused to participate. Eventually, 976 eligible mother-child pairs enrolled in the cohort. After excluding mothers with multiple births, conceived via assisted reproductive techniques, without antenatal care information, or who initiated antenatal care later than 13 weeks of gestation, a subset of 893 mother-child pairs was included in the final analysis. One-hundred and twenty-eight respondents had some missing data in their variables. When compared to the cases with complete data, cases with missing data were more likely to have parents with less education. No significant differences were found with respect to maternal parity, pre-pregnancy BMI, GWG, and children’s weight between missing and non-missing data groups (Supplemental Table S1). Table 1 shows the characteristics of the mother-child pairs.

Correlation coefficients among pre-pregnancy BMI, GWG, and children’s weight are summarized in Supplemental Table S2. Pre-pregnancy BMI had positive effects on the children’s weight from birth to 18 months old, while GWG was only positively associated with the children’s birth weight and the weight at 1 month or 12 months postpartum. Changes in the children’s weight by different maternal parity, pre-pregnancy BMI, and GWG categories are presented in Table 2. At birth, neonates of multiparas were heavier than neonates of primiparas. The differences in weight between infants of multiparas and primiparas still remained but had decreased at 1 month and 3 months of age, while this difference had disappeared at the age of 6 months. In contrast, the infants of multiparas were lighter than infants of primiparas after 6 months postpartum and this difference lasted to 18 months postpartum. The neonates of mothers who were underweight and gained less than recommended had lower mean birth weight than those of mothers with overweight or obesity. Weak positive associations were found in different GWG groups with children’s weight across time.

### 3.2. Latent Growth Curve Model

**Figure 1:**
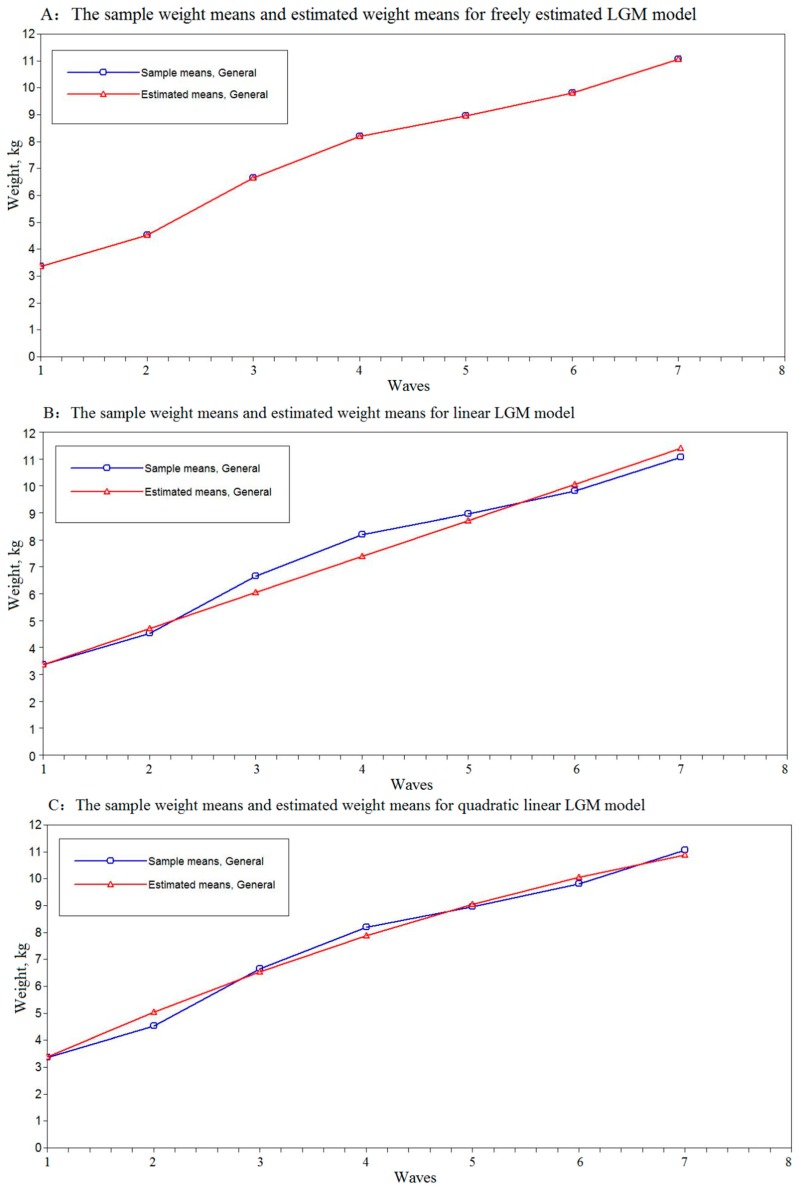
The weight means and estimated weight means of the children in unconditional freely estimated, linear, quadratic latent growth curve models. The child weight appears on the vertical axis (y) and the horizontal axis (x) represents the seven-time points; the axes should be continuous, not broken. The blue line represents the sample means and the red line represents the estimated means of the LGCMs. Panel (**A**) displays weight means and estimated weight means of the children in the unconditional freely estimated LGCM. Panel (**B**) displays weight means and estimated weight means of the children in the unconditional linear LGCM. Panel (**C**) displays weight means and estimated weight means of the children in the unconditional quadratic LGCM.

Table 3 presents the unconditional LGCM parameter estimates for the trajectories of children’s weight aged 0–18 months using LGCMs. Our results showed a substantively appreciable enhancement in model fit in freely estimated LGCM than the quadratic or linear models (Figure 1). Therefore, freely estimated LGCM was used to describe the trajectory of weight in early childhood. The estimate of intercept was 3.35 (*p* < 0.01), showing the initial level of the birth weight was 3.35 kg, consistent with the children’s mean birth weight. The estimate of the slope was 1.17 kg per wave (*p* < 0.01), showing a typical increase in the average rate of change in children’s weight. The free loadings of the slope were 0, 1.00, 2.83, 4.15, 4.80, 5.52, and 6.60, corresponding to the seven-time points.

**Figure 2:**
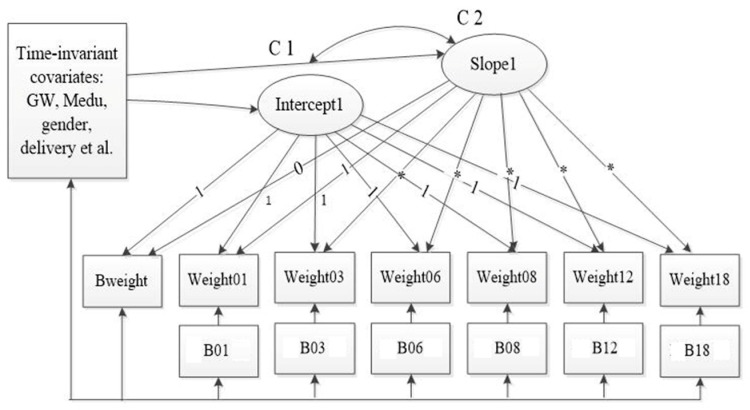
The measurement of LGCM with time-invariant and time-varying covariates for the trajectory of child weight. The time-varying covariates were the feeding practices such as whether engaged in any breastfeeding or introduced any formula during the study period. The time-invariant covariates were maternal age, child gender, pre-pregnancy BMI, GWG, paternal educational level, family income, multiparous, mode of delivery, physical exercise during pregnancy, maternal passive smoking during pregnancy, and GDM/hypertension. Abbreviations: Bweight, birth weight; weigth01–weight18, child weight at children’s 1, 3, 6, 8, 12, and 18 months old, B01–B18, whether engaged in breastfeeding during the study; GW, gestational week; Medu, maternal educational attachment; C1-C2, the correlation of the intercept1 and slope1.

Figure 2 shows the measurement of LGCM with covariates for the trajectory of child weight. Table 4 shows the estimates from the conditioned freely estimated LGCM. The model fitness was improved after adding the primary predictors and covariates incrementally. The intercept and slope factors remained statistically significant. Both maternal pre-conception BMI (β = 0.033, *p* < 0.01) and GWG (β = 0.014, *p* < 0.01) played important roles in the initial status of children’s weight, but didn’t have effects on the slope of the trajectory of weight change. Women who were multiparous had offspring with a higher birth weight (β = 0.110, *p* = 0.01), but had negative effects on the rate of weight change (β = −0.034, *p* = 0.01). What is more, these findings remained consistent and steady on the multiple imputation analyses. Child gender and gestational weeks were not only associated with the birth weight but also affected the rate of childhood weight change. Females had a lower mean birth weight than males and presented a slower increase in the rate of weight change. Additionally, children who had a longer gestational period were more likely to have large body size at birth, but presented slower rates of weight increase than their counterparts.

## 4. Discussion

This study indicated that model estimates under freely estimated LGCM can provide a favorable reflection of the growth trajectory of child weight aged 0–18 months. Our results documented that after taking account of the covariates, multiparous pregnancy was associated with higher birth weight and contributed to a slower weight-growth velocity. Maternal pre-pregnancy BMI and GWG had effects on the initial status of children’s weight, but showed no association with the future weight change in early childhood.

In line with previous studies [27,28], our findings showed that higher pregravid BMI and GWG had the potentials to contribute to an increase in children’s birth weight. Recently, a study compared the association of maternal pre-pregnancy and postpartum BMI Z scores with the child SD scores of weight and BMI at birth, 5 months, 12 months, and 7 years of age, and suggested that the differences among the associations were strong at birth but declined with child aging [29], which was consistent with our results. The effects of pre-pregnancy BMI and GWG declined with advancing age of the child, suggested that the lasting effects of this environment declined over time.

Consistent with our findings, a recent study suggested that offspring from primiparas have lower fetal but higher infant growth rates and higher risks of childhood overweight [15]. In another prospective cohort including 1335 infants indicated that infants of primiparas had strong catch-up growth, and from 12 months onward these infants were heavier compared with infants of multiparas [30]. Nolwenn and his colleagues reported a positive association between multiparous pregnancy and offspring birth weight, and this effect reduced with the advancing age of the infant [16]. However, different from the current study, no significant difference was found in the weight velocities in their study, which might result from the short follow-up period, as they only evaluated this association at the age of 1 month and 3 months. The results of the current study support that obesity risk can be passed from one generation to the next and maternal obesity, excessive gestational weight gain, and maternal parity can have adverse influences on later offspring weight status and rate of the weight change during early childhood. Although the observed effect estimates of these factors are small, they are mainly of interest from a child weight developmental perspective. Previous studies have shown that these childhood obesity risk factors may have lasting effects on adulthood obesity/overweight and are also related to the development of cardiovascular disease in later life [31,32]. It has therefore been recommended that achieving a healthy weight before pre-conception and a healthy weight gain during pregnancy may contribute to obesity and negative health outcomes prevention in childhood [33]. However, there is limited evidence for the effectiveness of interventions that target gestational or maternal weight gain on offspring obesity [34]. Higher pregravid BMI and GWG, as the modifiable risk factors, highlight the importance of public health implications that even small benefits are achievable in terms of preventing childhood overweight or obesity [35,36].

The mechanisms among pre-pregnancy BMI and GWG with early childhood weight are not clear. Increased pre-pregnancy BMI and GWG are thought to affect the children’s weight through regulatory changes in the specific intrauterine effects (increased insulin resistance, fetal glucose exposure, and low-grade systemic inflammation) [8], and sharing the same susceptibility genes of obesity [37]. Besides this, the amounts that children eat as well as food choices and eating behaviors in the early childhood were greatly influenced by their parents, and children may engage in the same unhealthy lifestyles as their mothers with obesity, such as intake of high-energy, low-nutrient foods, increased sedentary behavior, and deficient physical activity [38]. These pathways result in positive effects on promoting weight gain of offspring at birth, and even in later life. Similarly, the exact mechanisms linking maternal parity with child weight are still not completely understood. The spiral arteries, which provide maternal blood to the placenta, are remodeled during the first pregnancy. Moreover, these remodeled maternal vascular structures might offer a more favorable environment for placental development and fetal nutrition for multiparous pregnancies [39]. Additionally, the maternal metabolic and hormonal environments in multiparous women might differ from those in nulliparous women and these differences can also affect the fetal development [40]. The strengths of this study included its well-established longitudinal cohort, and its community-based sample, while most previous studies concerning this topic were based on cross-sectional or retrospective designs. Detailed, repeatedly measured, birth and childhood growth characteristics were available in the present study. To our best knowledge, this is the first longitudinal study in China investigating the contributions of pregravid BMI, GWG, and maternal parity to the development of children’s weight and its rate of change by using the LGCMs. Apart from maternal pre-pregnancy weight, which was based on the record of the maternal card, data for weight were all measured by the standardized techniques. Previous studies have demonstrated good validity and a strong correlation of the first antenatal visit weight and pre-pregnancy weight (*r* = 0.95, *p* < 0.01) [41].

Some attention should be paid when explaining the results. Due to the limitations of manpower and financial resources, we could not afford to conduct our study on a larger scale, limiting the external validity of our findings to all Chinese children. Second, although child weight is a direct measure to reflect the development of children, data on other anthropometric measures such as body composition were not available at regular check-ups in communities. Overweight and obesity are always predicted by anthropometric measures such as weight-for-length and BMI in infancy [42], whereas we only measured children’s weight, which was less useful for assessing the risk of fatness in longitudinal studies [43] and reduced the comparability of our results with other studies that used weight-for-length or BMI. Lastly, there are still several potential factors, such as child daily diet and physical activities that were not available, which might have influences on our findings. Therefore, in a future study, we will consider the diet quality and level of physical activity of children to address the potential influence on later childhood weight.

## 5. Conclusions

In summary, the implication of our results demonstrates that multiparous pregnancy, higher pre-pregnancy BMI, and GWG are associated with higher later offspring weight status, while only multiparous pregnancy contributes to a slower weight velocity during the first 18 months of life. Further well-designed and continued follow-up studies are needed to explore underlying mechanisms and clinical implications.

## Figures and Tables

**Table 1:** Characteristics of the mother-child pairs.

Variable	Value
Male, No. (%)	469 (52.9)
Maternal education, No. (%)	
Junior or below	31 (3.5)
High school	110 (12.5)
College or above	742 (84.0)
Paternal education, No. (%)	
Junior or below	29 (3.3)
High school	103 (11.7)
College or above	752 (85.0)
Family income, mean (SD), yuan	
≤5000	486 (56.0)
5001–10,000	346 (39.9)
≥10,001	36 (4.1)
Pre-pregnancy BMI group, mean (SD), kg/m^2^	
Underweight	169 (18.9)
Normal Weight	584 (65.4)
Overweight	107 (12.0)
Obesity	33 (3.7)
GWG group, mean (SD), kg	
Inadequate	186 (20.9)
Adequate	412(46.3)
Excessive	292(32.8)
Multiparous, No. (%)	266 (29.8)
Maternal age, mean (SD), years	29.9 (3.9)
Gestational week, median (IQR), weeks	39.0 (1.4)
Pre-pregnancy-BMI, mean (SD), kg/m^2^	21.3 (3.0)
Birth weight, mean (SD), kg	3.4 (0.4)
Weight at 1-month-old, mean (SD), kg	4.5 (0.6)
Weight at 3 months old, mean (SD), kg	6.7 (0.8)
Weight at 6 months old, mean (SD), kg	8.2 (0.9)
Weight at 8 months old, mean (SD), kg	8.9 (1.0)
Weight at 12 months old, mean (SD), kg	9.8 (1.0)
Weight at 18 months old, mean (SD), kg	11.1 (1.2)

Abbreviation: GWG, gestational weight gain; SD, standard deviation; IQR, interquartile range. Owing to missing data, the number of cases does not always sum to 893.

**Table 2:** Levels of child weight for different pre-pregnancy BMI groups and GWG groups.

Variable	Birth Weight		1 Month Old		3 Month Old		6 Month Old		8 Month Old		12 Month Old		18 Month Old	
	Mean (SD)	*p* ^1^	Mean (SD)	*p* ^1^	Mean (SD)	*p* ^1^	Mean (SD)	*p* ^1^	Mean (SD)	*p* ^1^	Mean (SD)	*p* ^1^	Mean (SD)	*p* ^1^
Weight, mean (SD), kg														
Total	3.35 (0.44)		4.53 (0.56)		6.66 (0.76)		8.19 (0.90)		8.94 (0.98)		9.80 (1.04)		11.07 (1.23)	
Maternal Parity					F = 0.26, *p* ^2^ = 0.61									
Primiparous	3.32 (0.46)	0.10	4.51(0.56)	0.07	6.64(0.76)	0.31	8.19(0.89)	0.92	8.97(0.95)	0.35	9.81(1.02)	0.60	11.08(1.22)	0.75
Multiparous	3.39 (0.41)		4.58(0.56)		6.70(0.75)		8.19(0.95)		8.90(1.06)		9.76(1.09)		11.05(1.28)	
Pre-pregnancy-BMI group, mean (SD), (kg/m^2^)					F = 22.37, *p* ^2^ < 0.01									
Underweight	3.21 (0.37)	<0.01	4.44 (0.49)	0.05	6.52 (0.71)	0.03	8.08 (0.84)	0.03	8.85 (0.91)	0.19	9.74 (1.01)	0.02	10.95 (1.14)	0.02
Normal Weight	3.35 (0.44)		4.53 (0.56)		6.67 (0.76)		8.18 (0.88)		8.94 (0.96)		9.75 (0.99)		11.03 (1.22)	
Overweight	3.50 (0.49)		4.62 (0.63)		6.75 (0.86)		8.32 (1.02)		9.09 (1.17)		10.01 (1.27)		11.36 (1.43)	
Obesity	3.60 (0.51)		4.64 (0.62)		6.85 (0.75)		8.52 (1.01)		9.12 (1.02)		10.15 (1.07)		11.47 (1.26)	
GWG group, mean (SD), kg					F = 4.37, *p* ^2^ = 0.01									
Inadequate	3.21 (0.45)	<0.01	4.46 (0.56)	0.04	6.56 (0.78)	0.12	8.10 (0.91)	0.31	8.78 (0.98)	0.06	9.60 (0.97)	0.01	11.01 (1.26)	0.10
Adequate	3.35 (0.40)		4.52 (0.52)		6.67 (0.75)		8.20 (0.91)		8.97 (1.00)		9.81 (1.05)		11.08 (1.27)	
Excessive	3.44 (0.48)		4.59 (0.61)		6.70 (0.74)		8.23 (0.89)		9.00 (0.94)		9.91 (1.05)		11.11 (1.20)	

Abbreviations: GWG, gestational weight gain, BMI, body mass index. ^1^ *p* value was calculated through one-way ANOVA or *t*-tests. ^2^ *p* was calculated through one-way repeated measures ANOVA.

**Table 3:** Parameter estimates of unconditional LGCMs for the trajectories of child weight aged 0–18 months old.

The Trajectory of Child Weight	Parameters	Coefficients	Z-Value	The Goodness of Fit Indices
Unconditional linear LGCM	Intercept	3.36 ***	219.02	$\chi^{2}\left(21\right)=1946.34$, *p*< 0.001, CFI = 0.21, TLI = 0.28, SRMR = 0.69; RMSEA = 0.31 (0.30–0.32)
	slope	1.34 ***	150.28	
Quadratic LGCM	Intercept	3.38 ***	204.59	$\chi^{2}\left(21\right)=1137.06$, *p*< 0.001, CFI = 0.54, TLI = 0.49, SRMR = 0.44; RMSEA = 0.26 (0.24–0.27)
	slope	1.75 ***	94.86	
	Quadratic slope	−0.08 ***	−25.09	
Conditional LGCM	Intercept	3.35 ***	225.11	$\chi^{2}\left(18\right)=213.98$, *p*< 0.001, CFI = 0.92, TLI = 0.91, SRMR = 0.19; RMSEA = 0.11 (0.10–0.12)
	slope	1.17 ***	80.71	
Freely estimated LGCM with multiple imputation data	Intercept	3.35 ***	225.33	$\chi^{2}\left(42\right)=$ 576.29, *p* < 0.001, CFI = 0.84, TLI = 0.83, SRMR = 0.18; RMSEA = 0.12 (0.11–0.13)
	slope	1.17 ***	80.86	

*** represents *p* < 0.001. Abbreviation: LGCM, latent growth curves model; TLI, Tucker-Lewis index; CFI, comparative fit index; SRMR, standardized root mean square residual; RMSEA, root mean square error of approximation.

**Table 4:** Estimates of the intercept and slope of the trajectory of child weight for the LGCMs.

Characteristic	Intercept		Slope		Intercept ^1^		Slope ^1^	
	*β*	*p*	*β*	*p*	*β*	*p*	*β*	*p*
Time-invariant covariates								
Gender	−0.165	<0.01	−0.070	<0.01	−0.150	<0.01	−0.079	<0.01
Gestational week	0.135	<0.01	−0.014	<0.01	0.136	<0.01	−0.016	<0.01
Pre-pregnancy BMI	0.034	<0.01	0.000	0.87	0.030	<0.01	0.001	0.78
GWG	0.014	<0.01	−0.002	0.10	0.014	<0.01	−0.001	0.28
Maternal age	−0.001	0.75	0.002	0.16	−0.001	0.82	0.002	0.27
Maternal education	−0.021	0.42	0.005	0.61	−0.017	0.50	0.007	0.51
Family income	0.005	0.81	−0.002	0.80	0.005	0.80	−0.003	0.77
Multiparous	0.103	<0.01	−0.028	0.02	0.110	0.01	−0.032	0.02
Time-varying covariates	Estimates		*p*		Estimates		*p*	
Breastfeeding01 → Weight01	0.160		0.04		0.018		0.82	
Formula01 → Weight01	0.013		0.71		0.013		0.70	
Breastfeeding03 → Weight03	−0.008		0.91		-0.026		0.69	
Formula03 → Weight03	−0.011		0.83		−0.009		0.85	
Breastfeeding06 → Weight06	0.006		0.91		0.026		0.61	
Formula06 → Weight06	−0.094		0.04		−0.074		0.09	
Breastfeeding08 → Weight08	−0.012		0.82		0.010		0.83	
Formula08 → Weight08	−0.097		0.05		−0.097		0.15	
Breastfeeding12 → Weight12	0.053		0.43		0.027		0.67	
Formula12 → Weight12	0.086		0.24		0.073		0.38	
Breastfeeding18 → Weight18	0.026		0.23		0.015		0.75	
Formula18 → Weight18	0.054		0.42		0.056		0.56	
Fit indices								
AIC	9441.205				11666.693			
BIC	9641.775				11882.449			
RMSER	0.047				0.049			
CFI	0.938				0.921			
TLI	0.922				0.905			
SRMR	0.051				0.058			
P (chi-square test)	<0.01				<0.01			

Abbreviations: GWG, gestational weight gain; weight01–weight18, child weight at the ages of 1 to 18 months; formula01–formula18, child formula at the ages of 1 to 18 months, breastfeeding01–breastfeeding18, child breastfeeding status at the ages of 1 to 18 month. ^1^ presented the data analyses with multiple imputation data.

## Supplementary Materials

The following are available online at https://www.mdpi.com/1660-4601/16/7/1110/s1, Table S1: Non-response analysis for missing data group and non-missing group, Table S2: Correlation coefficients between maternal pre-pregnancy BMI, GWG, and child weight from birth to 18 months old.

## References

[1] Gao X., Yan Y., Xiang S., Zeng G.Y., Liu S.P., Sha T.T., He Q., Li H.Y., Tan S., Chen C. (2017). The mutual effect of pre-pregnancy body mass index, waist circumference and gestational weight gain on obesity-related adverse pregnancy outcomes: A birth cohort study. PLoS ONE.

[2] Flegal K.M., Carroll M.D., Kit B.K., Ogden C.L. (2012). Prevalence of obesity and trends in the distribution of body mass index among US adults, 1999–2010. JAMA.

[3] Bendixen H., Holst C., Sorensen T.I., Ogden C.L. (2004). Major increase in prevalence of overweight and obesity between 1987 and 2001 among Danish adults. Obes. Res..

[4] Goldstein R.F., Abell S.K., Ranasinha S., Misso M., Boyle J.A., Black M.H., Li N., Hu G., Corrado F., Rode L. (2017). Association of Gestational Weight Gain with Maternal and Infant Outcomes: A Systematic Review and Meta-analysis. JAMA.

[5] Soltani H., Lipoeto N.I., Fair F.J., Kilner K., Yusrawati Y. (2017). Pre-pregnancy body mass index and gestational weight gain and their effects on pregnancy and birth outcomes: A cohort study in West Sumatra, Indonesia. BMC Womens Health.

[6] Nelson S.M., Matthews P., Poston L. (2010). Maternal metabolism and obesity: modifiable determinants of pregnancy outcome. Hum. Reprod. Update.

[7] Gaillard R., Rifas-Shiman S.L., Perng W., Oken E., Gillman M.W. (2016). Maternal inflammation during pregnancy and childhood adiposity. Obesity.

[8] Gaillard R. (2015). Maternal obesity during pregnancy and cardiovascular development and disease in the offspring. Eur. J. Epidemiol..

[9] Fraser A., Tilling K., Macdonald-Wallis C., Sattar N., Brion M.J., Benfield L., Ness A., Deanfield J., Hingorani A., Nelson S.M. (2010). Association of maternal weight gain in pregnancy with offspring obesity and metabolic and vascular traits in childhood. Circulation.

[10] Zhang C., Hediger M.L., Albert P.S., Grewal J., Sciscione A., Grobman W.A., Wing D.A., Newman R.B., Wapner R., D’Alton M.E. (2018). Association of Maternal Obesity with Longitudinal Ultrasonographic Measures of Fetal Growth: Findings from the NICHD Fetal Growth Studies-Singletons. JAMA Pediatr..

[11] Kim J., Peterson K.E., Scanlon K.S., Fitzmaurice G.M., Must A., Oken E., Rifas-Shiman S.L., Rich-Edwards J.W., Gillman M.W. (2006). Trends in overweight from 1980 through 2001 among preschool-aged children enrolled in a health maintenance organization. Obesity.

[12] Denison F.C., Roberts K.A., Barr S.M., Norman J.E. (2010). Obesity, pregnancy, inflammation, and vascular function. Reproduction.

[13] Shin D., Chung H., Weatherspoon L., Song W.O. (2014). Validity of prepregnancy weight status estimated from self-reported height and weight. Matern. Child Health J..

[14] Weng S.F., Redsell S.A., Swift J.A., Yang M., Glazebrook C.P. (2012). Systematic review and meta-analyses of risk factors for childhood overweight identifiable during infancy. Arch. Dis. Child..

[15] Gaillard R., Rurangirwa A.A., Williams M.A., Hofman A., Mackenbach J.P., Franco O.H., Steegers E.A.P., Jaddoe V.W.V. (2014). Maternal parity, fetal and childhood growth, and cardiometabolic risk factors. Hypertension.

[16] Regnault N., Botton J., Forhan A., Hankard R., Thiebaugeorges O., Hillier T.A., Kaminski M., Heude B., Charles M.A. (2010). Determinants of early ponderal and statural growth in full-term infants in the EDEN mother-child cohort study. Am. J. Clin. Nutr..

[17] Reilly J.J., Armstrong J., Dorosty A.R., Emmett P.M., Ness A., Rogers I., Steer C., Sherriff A., Avon Longitudinal Study of Parents and Children Study Team (2005). Early life risk factors for obesity in childhood: Cohort study. BMJ.

[18] World Health Organization (2016). Report of the Commission on Ending Childhood Obesity.

[19] Ogden C.L., Carroll M.D., Lawman H.G., Fryar C.D., Kruszon-Moran D., Kit B.K., Flegal K.M. (2016). Trends in Obesity Prevalence Among Children and Adolescents in the United States, 1988–1994 Through 2013–2014. JAMA.

[20] Blake-Lamb T.L., Locks L.M., Perkins M.E., Woo Baidal J.A., Cheng E.R., Taveras E.M. (2016). Interventions for Childhood Obesity in the First 1,000 Days A Systematic Review. Am. J. Prev. Med..

[21] Sha T., Yan Y., Gao X., Xiang S.T., Zeng G.Y., Liu S.P., He Q. (2017). Association between Sleep and Body Weight: A Panel Data Model Based on a Retrospective Longitudinal Cohort of Chinese Infants. Int. J. Environ. Res. Public Health.

[22] Institute of Medicine (2009). Weight Gain During Pregnancy: Reexamining the Guidelines.

[23] McPhie S., Skouteris H., Mattick R.P., Wilson J., Honan I., Allsop S., Burns L., Elliott E., Teague S., Olsson C.A. (2017). Weight in the First Year of Life: Associations with Maternal Pre-pregnancy Body Mass Index and Gestational Weight Gain-Findings from a Longitudinal Pregnancy Cohort. Am. J. Perinatol..

[24] Yan J. (2015). Maternal pre-pregnancy BMI, gestational weight gain, and infant birth weight: A within-family analysis in the United States. Econ. Hum. Biol..

[25] Kline R.B. (2010). Principles and Practice of Structural Equation Modeling.

[26] Wu W., West S.G., Taylor A.B. (2009). Evaluating model fit for growth curve models: Integration of fit indices from SEM and MLM frameworks. Psychol. Methods.

[27] Ensenauer R., Chmitorz A., Riedel C., Fenske N., Hauner H., Nennstiel-Ratzel U., von Kries R. (2013). Effects of suboptimal or excessive gestational weight gain on childhood overweight and abdominal adiposity: Results from a retrospective cohort study. Int. J. Obes..

[28] Robinson C.A., Cohen A.K., Rehkopf D.H., Deardorff J., Ritchie L., Jayaweera R.T., Coyle J.R., Abrams B. (2014). Pregnancy and post-delivery maternal weight changes and overweight in preschool children. Prev. Med..

[29] Sorensen T., Ajslev T.A., Angquist L., Morgen C.S., Ciuchi I.G., Smith G.D. (2016). Comparison of associations of maternal peri-pregnancy and paternal anthropometrics with child anthropometrics from birth through age 7 y assessed in the Danish National Birth Cohort. Am. J. Clin. Nutr..

[30] Ong K.K., Preece M.A., Emmett P.M., Ahmed M.L., Dunger D.B., ALSPAC Study Team (2002). Size at birth and early childhood growth in relation to maternal smoking, parity and infant breast-feeding: Longitudinal birth cohort study and analysis. Pediatr. Res..

[31] Juhola J., Magnussen C.G., Viikari J.S., Kahonen M., Hutri-Kahonen N., Jula A., Lehtimaki T., Akerblom H.K., Pietikainen M., Laitinen T. (2011). Tracking of serum lipid levels, blood pressure, and BMI from childhood to adulthood: The Cardiovascular Risk in Young Finns Study. J. Pediatr..

[32] Franks P.W., Hanson R.L., Knowler W.C., Sievers M.L., Bennett P.H., Looker H.C. (2010). Childhood obesity, other cardiovascular risk factors, and premature death. N. Engl. J. Med..

[33] Olson C.M., Strawderman M.S., Dennison B.A. (2009). Maternal weight gain during pregnancy and child weight at age 3 years. Matern. Child Health J..

[34] Hanson M., Barker M., Dodd J.M., Kumanyika S., Norris S., Steegers E., Stephenson J., Thangaratinam S., Yang H. (2017). Interventions to prevent maternal obesity before conception, during pregnancy, and post partum. Lancet Diabetes Endocrinol..

[35] Macdonald-Wallis C., Tilling K., Fraser A., Nelson S.M., Lawlor D.A. (2013). Gestational weight gain as a risk factor for hypertensive disorders of pregnancy. Am. J. Obstet. Gynecol..

[36] Carreno C.A., Clifton R.G., Hauth J.C., Myatt L., Roberts J.M., Spong C.Y., Varner M.W., Thorp J.M., Mercer B.M., Peaceman A.M. (2012). Excessive early gestational weight gain and risk of gestational diabetes mellitus in nulliparous women. Obstet. Gynecol..

[37] Kral J.G., Biron S., Simard S., Hould F.S., Lebel S., Marceau S., Marceau P. (2006). Large maternal weight loss from obesity surgery prevents transmission of obesity to children who were followed for 2 to 18 years. Pediatrics.

[38] O’Connor S.G., Koprowski C., Dzubur E., Leventhal A.M., Huh J., Dunton G.F. (2017). Differences in Mothers’ and Children’s Dietary Intake during Physical and Sedentary Activities: An Ecological Momentary Assessment Study. J. Acad. Nutr. Diet..

[39] Zalud I., Shaha S. (2008). Three-dimensional sonography of the placental and uterine spiral vasculature: Influence of maternal age and parity. J. Clin. Ultrasound..

[40] Gunderson E.P., Lewis C.E., Murtaugh M.A., Quesenberry C.P., West D.S., Sidney S. (2004). Long-term plasma lipid changes associated with a first birth: The Coronary Artery Risk Development in Young Adults study. Am. J. Epidemiol..

[41] Phelan S., Phipps M.G., Abrams B., Darroch F., Schaffner A., Wing R.R. (2011). Randomized trial of a behavioral intervention to prevent excessive gestational weight gain: The Fit for Delivery Study. Am. J. Clin. Nutr..

[42] Bell K.A., Wagner C.L., Perng W., Feldman H.A., Shypailo R.J., Belfort M.B. (2018). Validity of body mass index as a measure of adiposity in infancy. J. Pediatr..

[43] Cole T.J., Faith M.S., Pietrobelli A., Heo M. (2005). What is the best measure of adiposity change in growing children: BMI, BMI %, BMI z-score or BMI centile?. Eur. J. Clin. Nutr..

