# Supplementary material for: Associations of Pre-Pregnancy BMI, Gestational Weight Gain and Maternal Parity with the Trajectory of Weight in Early Childhood: A Prospective Cohort Study
Source: Int J Environ Res Public Health. 2019 Mar 28;16(7):1110. doi: 10.3390/ijerph16071110 (PMC6480263; doi:10.3390/ijerph16071110)
Supplement: Supplementary file 1 [file ijerph-16-01110-s001.pdf]

Table S1. Non-response analysis for missing data group and non-missing group

Variable	Non-missing (n=128)	Missing (n=765)	Statistics	P
Male, No. (%)	400(52.7)	69(54.3)	0.1	0.73
Maternal education, No. (%)				
Junior or below	28(3.7)	3(2.4)		
High school	86(11.4)	24(19.1)	6.2	0.04
College or above	643(84.9)	99(78.6)		
Paternal education, No. (%)				
Junior or below	24(3.2)	5(3.9)		
High school	75(9.9)	28(22.1)	16.1	<0.01
College or above	658(86.9)	94(74.0)		
Family income, mean (SD), yuan				
≤5000	415(55.9)	71(56.8)		
5001-1000	298(40.1)	48(38.4)	0.2	0.89
≥10001	30(4.0)	6(4.8)		
Pre-pregnancy BMI group, mean (SD), kg/m2				
<18.5	148(19.4)	21(16.4)		
18.5-23.9	497(65.0)	87(68.0)	4.1	0.25
24-27.9	95(12.4)	12(9.4)		
>=28	25(3.3)	8(6.3)		
GWG group, mean (SD),kg				
Inadequate	160(21.0)	26(20.3)		
Adequate	350(45.9)	62(48.4)	0.3	0.87
Excessive	252(33.1)	40(31.2)		
Multiparous, No. (%)	230(30.1)	36(28.1)	0.2	0.66
Maternal age, mean (SD), year	29.8(3.8)	30.5(4.5)	-1.6	0.11
Gestational week, median (IQR), week	39.0(1.4)	38.9(1.5)	0.8	0.45
Pre-pregnancy-BMI, mean (SD), kg/m2	21.2(3.0)	21.4(3.2)	-0.6	0.56
Birth weight, mean (SD),kg	3.4(0.6)	3.3(0.5)	0.6	0.52
weight01, mean (SD),kg	4.5(0.6)	4.5(0.6)	0.5	0.59
weight03, mean (SD),kg	6.7(0.8)	6.7(0.7)	-1.0	0.31
weight06, mean (SD),kg	8.2(0.9)	8.2(0.8)	-0.2	0.89
weight08, mean (SD),kg	8.9(1.0)	9.0 (1.0)	-0.7	0.52
weight12, mean (SD),kg	9.9(3.2)	9.7(0.9)	1.6	0.10
weight18, mean (SD),kg	11.1(1.2)	10.9(1.4)	0.8	0.45

Abbreviation: GWG, gestational weight gain; weight01-weight18, child weight at the ages of 1 to 18 months.

Table S2. Correlation coefficients between maternal prepregnancy BMI, GWG and child weight from birth to 18 months old

	Prepregnancy BMI	GWG	Birth weight	Weight01	Weight03	Weight06	Weight08	Weight12	Weight18
Prepregnancy BMI	1.000	-0.163***	0.211***	0.099**	0.094**	0.097**	0.082*	0.104***	0.126***
GWG	-0.163***	1.000	0.151***	0.082*	0.031	0.029	0.050	0.073*	-0.013
Birth-weight	0.211***	0.151***	1.000	0.663***	0.418***	0.369***	0.348***	0.305***	0.286***
weight01	0.099**	0.082**	0.663***	1.000	0.509***	0.428***	0.401***	0.330***	0.296***
weight03	0.094**	0.031	0.418***	0.509***	1.000	0.687***	0.658***	0.509***	0.487***
weight06	0.097**	0.029	0.369***	0.428***	0.687***	1.000	0.818***	0.681***	0.626***
weight08	0.082**	0.050	0.348***	0.401***	0.658***	0.818***	1.000	0.725***	0.672***
weight12	0.104***	0.073*	0.305***	0.330***	0.509***	0.681***	0.725***	1.000	0.722***
weight18	0.126***	-0.013	0.286***	0.296***	0.487***	0.626***	0.672***	0.722***	1.000

Abbreviation: GWG, gestational weight gain; weight01-weight18: child weight at the ages of 1 to 18 months

*P<0.05, **P<0.01, ***P<0.001.
